# Pharmacological enhancement of TFEB-mediated autophagy alleviated neuronal death in oxidative stress-induced Parkinson’s disease models

**DOI:** 10.1038/s41419-020-2322-6

**Published:** 2020-02-18

**Authors:** Xu-Xu Zhuang, Sheng-Fang Wang, Yuan Tan, Ju-Xian Song, Zhou Zhu, Zi-Ying Wang, Ming-Yue Wu, Cui-Zan Cai, Zhi-Jian Huang, Jie-Qiong Tan, Huan-Xing Su, Min Li, Jia-Hong Lu

**Affiliations:** 1 State Key Laboratory of Quality Research in Chinese Medicine, Institute of Chinese Medical Sciences, University of Macau, Macau, SAR China; 2 Mr. and Mrs. Ko Chi Ming Centre for Parkinson’s Disease Research, School of Chinese Medicine, Hong Kong Baptist University, Hong Kong, SAR China; 3 Medical College of Acupuncture-Moxibustion and Rehabilitation, Guangzhou University of Chinese Medicine, Guangzhou, China; 4 Center for Medical Genetics, School of Life Sciences, Central South University, Changsha, Hunan China

**Keywords:** Macroautophagy, Parkinson's disease, Cell death

## Abstract

Autophagy, a conserved cellular degradation and recycling process, can be enhanced by nutrient depletion, oxidative stress or other harmful conditions to maintain cell survival. 6-Hydroxydopamine/ascorbic acid (6-OHDA/AA) is commonly used to induce experimental Parkinson’s disease (PD) lesions by causing oxidative damage to dopaminergic neurons. Activation of autophagy has been observed in the 6-OHDA-induced PD models. However, the mechanism and exact role of autophagy activation in 6-OHDA PD model remain inconclusive. In this study, we report that autophagy was triggered via mucolipin 1/calcium/calcineurin/TFEB (transcription factor EB) pathway upon oxidative stress induced by 6-OHDA/AA. Interestingly, overexpression of TFEB alleviated 6-OHDA/AA toxicity. Moreover, autophagy enhancers, Torin1 (an mTOR-dependent TFEB/autophagy enhancer) and curcumin analog C1 (a TFEB-dependent and mTOR-independent autophagy enhancer), significantly rescued 6-OHDA/AA-induced cell death in SH-SY5Y cells, iPSC-derived DA neurons and mice nigral DA neurons. The behavioral abnormality of 6-OHDA/AA-treated mice can also be rescued by Torin 1 or C1 administration. The protective effects of Torin 1 and C1 can be blocked by autophagy inhibitors like chloroquine (CQ) or by knocking down autophagy-related genes TFEB and ATG5. Taken together, this study supports that TFEB-mediated autophagy is a survival mechanism during oxidative stress and pharmacological enhancement of this process is a neuroprotective strategy against oxidative stress-associated PD lesions.

## Introduction

Macroautophagy (hereafter referred to as autophagy), an intracellular bulk degradation process, plays an important role in degrading and recycling cytosolic long-lived proteins and damaged organelles via the lysosome. Autophagy operates constitutively at a basal level under normal conditions, and could be induced by the nutrient depletion, oxidative stress, or other harmful conditions^1,2^. As a survival mechanism for cells during extracellular and intracellular stress, impairment of autophagy has been linked to various diseases^3^, including neurodegenerative disorders like Parkinson’s disease (PD)^4^.

PD, featured by the loss of dopaminergic neurons in the substantia nigra pars compacta (SNc), is a prevalent neurodegenerative disorder among aging individuals. To date, the pathogenesis of nigral degeneration remains unclear. Increasing evidence revealed that reactive oxygen species (ROS) and defects in the clearance of abnormal protein aggregates are important factors underlying it^5,6^. Therefore, the role of autophagy in the degradation of damaged mitochondria and accumulated α-synuclein aggregates occurring in PD has generated interest in the development of therapeutic strategies^7–10^.

6-Hydroxydopamine (6-OHDA), a neurotoxin associated with mitochondrial damage and ROS such as hydrogen peroxide, superoxide, and hydroxyl radicals^11^, is commonly used to model PD both in vitro and in vivo^12,13^. In the presence of ascorbic acid (AA), the toxicity of 6-OHDA can be enhanced^14–16^. Changes of autophagy status have been reported in 6-OHDA-induced PD models^17–21^, however, the mechanisms and consequence of autophagy status changes have not been well explored. Recently, it was reported that a mild increase of ROS levels could activate mucolipin 1 (MCOLN1), a key calcium-conducting channel on the lysosome membrane, to initiate calcineurin-dependent activation of transcription factor EB (TFEB)^22^, which is identified as a master regulator of the autophagy–lysosome pathway (ALP)^23^. In turn, the TFEB-mediated induction of autophagy promotes clearance of damaged mitochondria and removal of excess ROS^22^. However, excessive ROS levels may cause lysosomal dysfunction and autophagic failure, and lead to cell death^22,24^. Similarly, the activation levels and induction time of autophagy also play critical role in the survival or death of cells^25–27^.

Activation of TFEB has been shown to alleviate neurodegeneration in several in vitro and in vivo models of neurodegenerative diseases through lysosomal function enhancement and autophagy induction^8,23,28^. In this study, we showed that autophagy is triggered through calcineurin-dependent TFEB activation to reduce neuronal death in 6-OHDA/AA-lesioned models of PD. The neuroprotective effects of autophagy against 6-OHDA/AA toxicity could be significantly improved by TFEB enhancers, such as Torin 1, a potent mammalian target of rapamycin (mTOR) inhibitor^29^, and C1, a novel curcumin analog binding to and activating TFEB independent of mTOR inhibition^30^. In addition, chemical or genetic approaches inhibiting ALP impairs the neuroprotection induced by Torin 1 or C1.

## Results

### TFEB is activated both in vitro and in vivo in the 6-OHDA/AA models

**Fig. 1:**
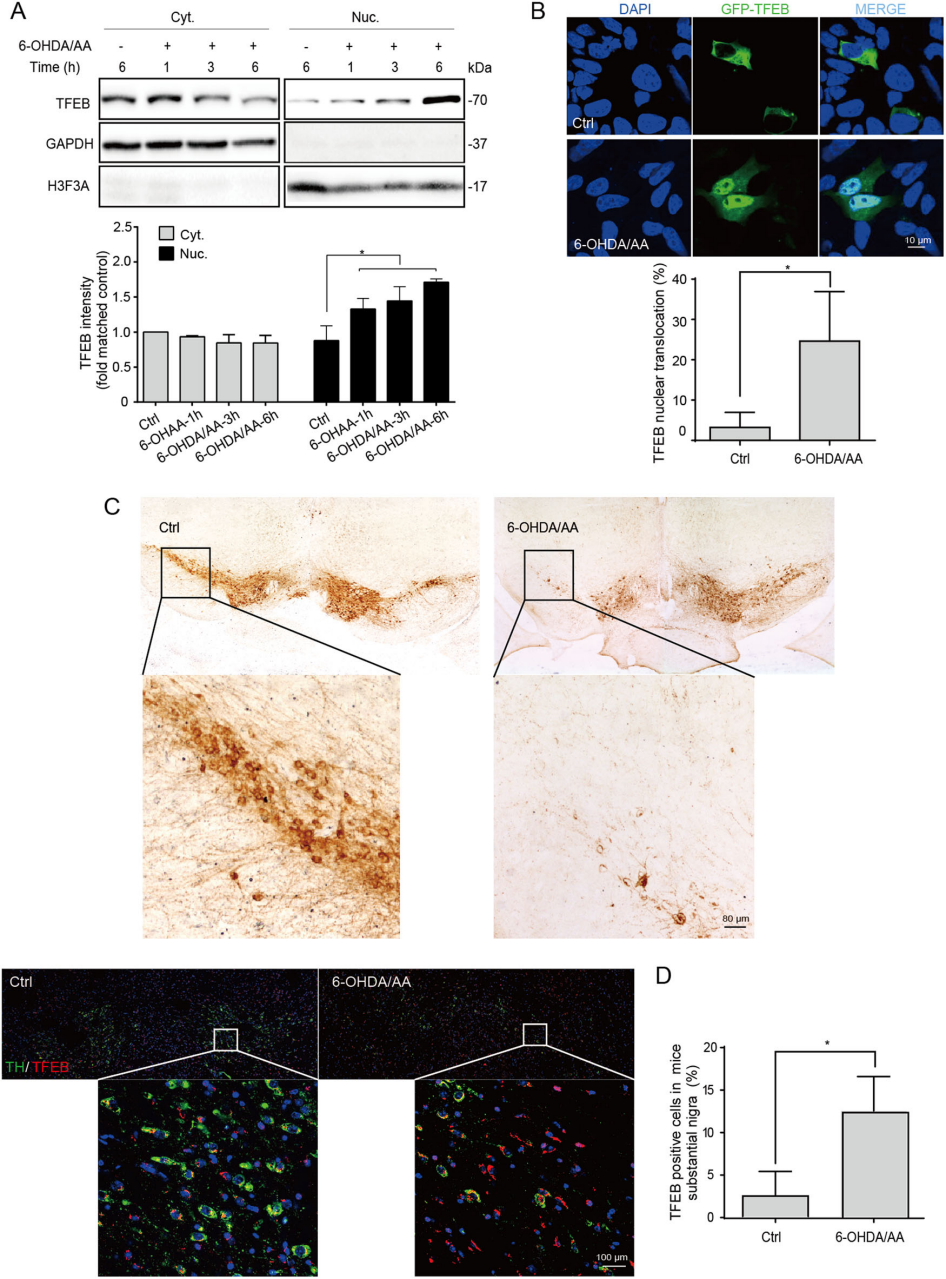
TFEB is activated both in vitro and in vivo in the 6-OHDA/AA models. **a** SH-SY5Y cells were treated with 20 μM 6-OHDA in culture medium containing 0.15% ascorbic acid (6-OHDA/AA) for 15 min, and then replaced with normal culture medium and incubated for indicated time points. The levels of endogenous TFEB in the cytosolic (Cyt.) and nuclear (Nuc.) fractions were detected by Western blot (*n* = 5). GAPDH and H3F3A were used as loading controls of the cytosolic and nuclear fractions, respectively. **b** SH-SY5Y cells were transiently transfected with TFEB-GFP for 24 h and then treated with 6-OHDA/AA) for 15 min, and then replaced with normal culture medium and incubated 6 h. The representative images showed intracellular localization of TFEB. At least 30 cells were randomly selected and analyzed in each treatment group (*n* = 3). **c** Representative images showed tyrosine hydroxylase (TH)-positive cells in the substantia nigra pars compacta (SNc) of mouse brain from control and 6-OHDA/AA groups. **d** The TFEB intracellular localization revealed by immunofluorescence staining with TFEB (red) and TH (green) in neurons of SNc (*n* = 6). The quantification results are shown as mean ± SD. *P < 0.05 vs. control (Ctrl) group.

As a transcription factor, phosphorylated TFEB is retained in the cytoplasm under normal conditions. Upon starvation or other stimulations, dephosphorylated TFEB translocates to the nucleus and subsequently initiates the transcription of genes related to autophagy and lysosomal biogenesis. We found the nuclear translocation of TFEB in SH-SY5Y cells treated with 6-OHDA/AA was increased in a time-dependent manner (Fig. 1a). Next, the cellular distribution of TFEB in SH-SY5Y cells transiently transfected with GFP-TFEB was detected by fluorescence imaging, namely, we detected the intensity of GFP-TFEB in the nuclei. The *y*-axis shows the percent of TFEB-GFP-transiently expressed cells with TFEB-nuclear localization. Consistently, 6-OHDA/AA significantly promoted GFP-TFEB-nuclear accumulation (Fig. 1b). We further checked the expression and nuclear localization of TFEB in *SNc* of C57 mice injected with 6-OHDA/AA in unilateral striatum for 21 days. Firstly, the tyrosine hydroxylase (TH) immunostaining was used to confirm the dopaminergic neuron degeneration in SNc. The mice treated with 6-OHDA/AA showed massive reduction in the number of TH-positive cells in the *SNc* compared to the non-lesioned side, indicating the successful establishment of PD injury (Fig. 1c). And then the activation of TFEB was detected by immunohistofluorescence. SNc neurons of mice in the model group show striking TFEB activation (Fig. 1d) indicated by the increasing nuclear translocation. Above results suggest that TFEB is activated in neuronal cells treated with 6-OHDA/AA.

### ALP is activated in 6-OHDA/AA models

**Fig. 2:**
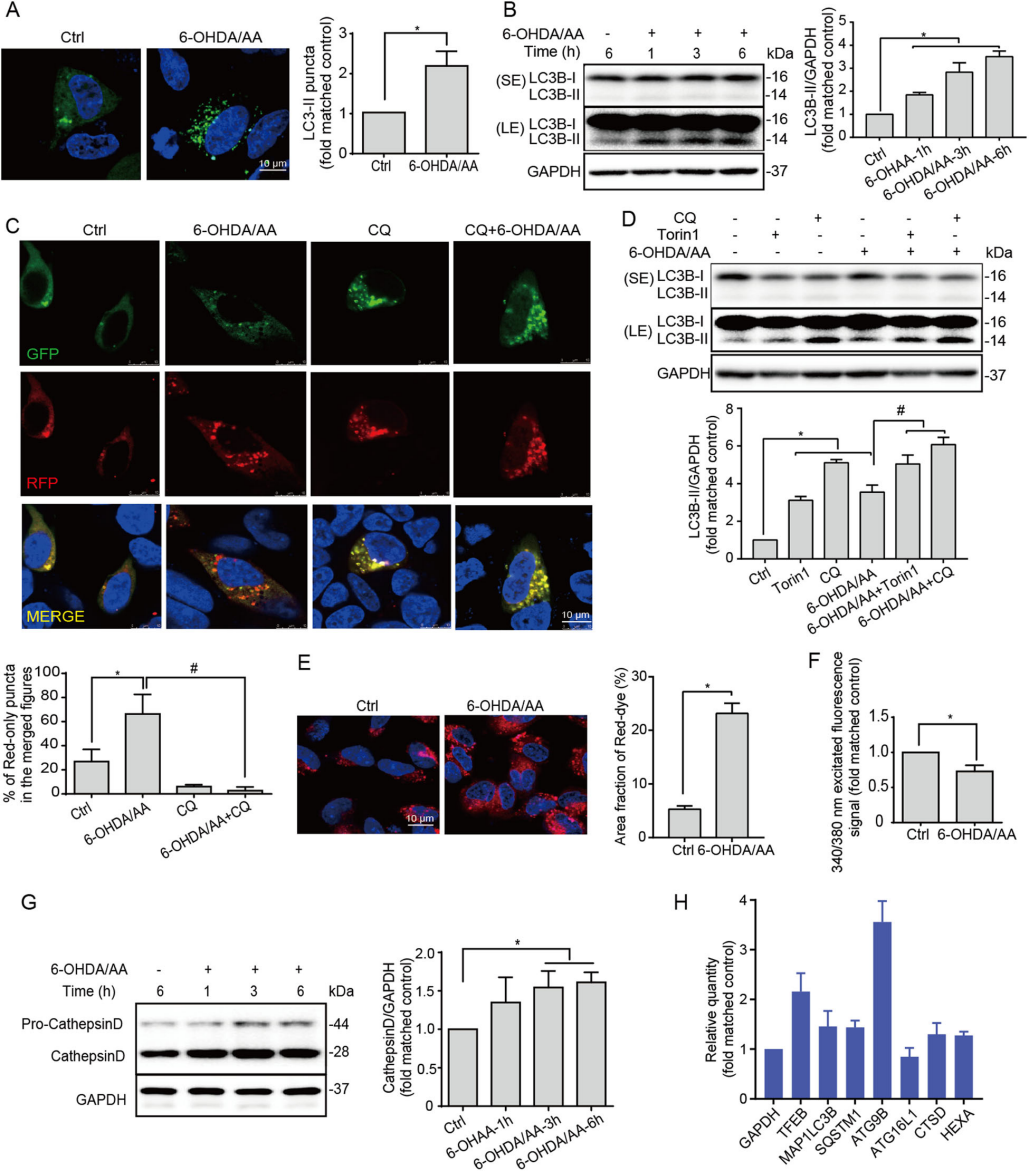
Autophagy–lysosome pathway (ALP) is activated in 6-OHDA/AA models. **a** SH-SY5Y cells transfected with GFP-LC3 construct for 24 h were treated with 20 μM 6-OHDA/AA for 15 min, and then the medium was replaced with normal culture medium and incubated for indicated time points. The total GFP-LC3 intensity (green) were measured by immunofluorescence. At least 30 cells were randomly selected and analyzed in each treatment group (*n* = 3). **b** SH-SY5Y cells were treated with 20 μM 6-OHDA/AA as described in panel (**a**) for indicated time points. The upper band showed the short-time exposure (LE) of LC3B while the middle one showed the long-time exposure (LE) of LC3B (*n* = 5). **c** SH-SY5Y cells were transfected with GFP-RFP-LC3 construct for 24 h and next treated with 20 μM 6-OHDA/AA with or without chloroquine (CQ, 15 μM) for 15 min, and then changed to normal cultured medium with or without CQ and incubated for 6 h. The fluorescence signals were checked under a confocal microscope. At least 30 cells were randomly selected and analyzed in each treatment group (*n* = 3). **d** SH-SY5Y cells were treated as described in panel (**c**). **e** SHSY5Y cells were treated with 20 μM 6-OHDA/AA as described in panel (**a**) for 6 h. After the treatment, cells were stained with LysoTracker Red DND-99 (50 nM) for 30 min and then were checked under a confocal microscope. **g** SHSY-5Y cells were stained with LysoSensor Yellow/Blue DND-160 (10 μM) for 20 min and detected with a plate reader at the excited fluorescence signal 340 and 380 nm (*n* = 5). **h** SH-SY5Y cells were treated with 6-OHDA/AA as described in panel (**a**) for indicated time points, and the cell lysate was collected and subjected to WB analysis (*n* = 5). **i** mRNA transcript abundance was assessed by real-time PCR using specific primers for the indicated genes (*n* = 5). Above quantifications are shown as mean ± SD. *P < 0.05 vs. Ctrl group; ^#^*P* < 0.05 vs. 6-OHDA/AA group.

TFEB is the master control of ALP, and we sought to know whether the ALP was also activated in this 6-OHDA/AA toxicity model. The expression of LC3B-II, an autophagosome marker reflecting autophagy activity, was detected in SH-SY5Y cells treated with 6-OHDA/AA at different time points. We observed an increase in the number of GFP-LC3 puncta in cells transfected with GFP-LC3 plasmid (Fig. 2a) and a time-dependent increase of LC3B-II by western blot (Fig. 2b). To further confirm that 6-OHDA/AA-induced autophagosome increase was due to autophagy activation instead of the impairment in autophagosome maturation, we used the GFP-RFP-LC3 probe to analyze the maturation process in SH-SY5Y cells following treatment with 6-OHDA/AA or chloroquine (CQ), an autophagy inhibitor impairing autophagosome fusion with lysosome^31^. Compared with control group, the number of the yellow puncta (early autophagosomes) and red puncta (mature autophagosomes) in the 6-OHDA/AA group were both increased, demonstrating the maturation of autophagosomes greatly increased upon 6-OHDA/AA treatment for 6 h, whereas the cotreatment with CQ group showed more significant blocked maturation compared the CQ group (Fig. 2c). Consistently, in the presence of CQ, the LC3B-II level was further increased compared with 6-OHDA/AA treatment alone (Figs. 2d and S1e), indicating an increase of autophagy flux rather than maturation blockage. The change of lysosome function was also measured in SH-SY5Y cells treated with 6-OHDA/AA at different time points. Firstly, we used LysoTracker Red DND-99 and LysoSensor Yellow/Blue DND-160 to detect the change of lysosomal pH. The significant increase in Red-dye accumulation (Fig. 2e) and reduction in 340/380 fluorescence ratio (Fig. 2f) indicated that 6-OHDA/AA greatly enhanced lysosomal acidification. Next, lysosomal enzyme Cathepsin D were detected by western blot. As expected, 6-OHDA/AA time-dependently increased the expression of Cathepsin D (Fig. 2g). Furthermore, at the gene expression level, 6-OHDA/AA increased the transcription of a series of ALP related genes in SH-SY5Y cells (Fig. 2h). Collectively, these results indicated that the ALP was quickly activated (within 6 h) in the 6-OHDA/AA model.

### TFEB-mediated autophagy is inducted in a calcium and calcineurin-dependent manner in 6-OHDA/AA models

**Fig. 3:**
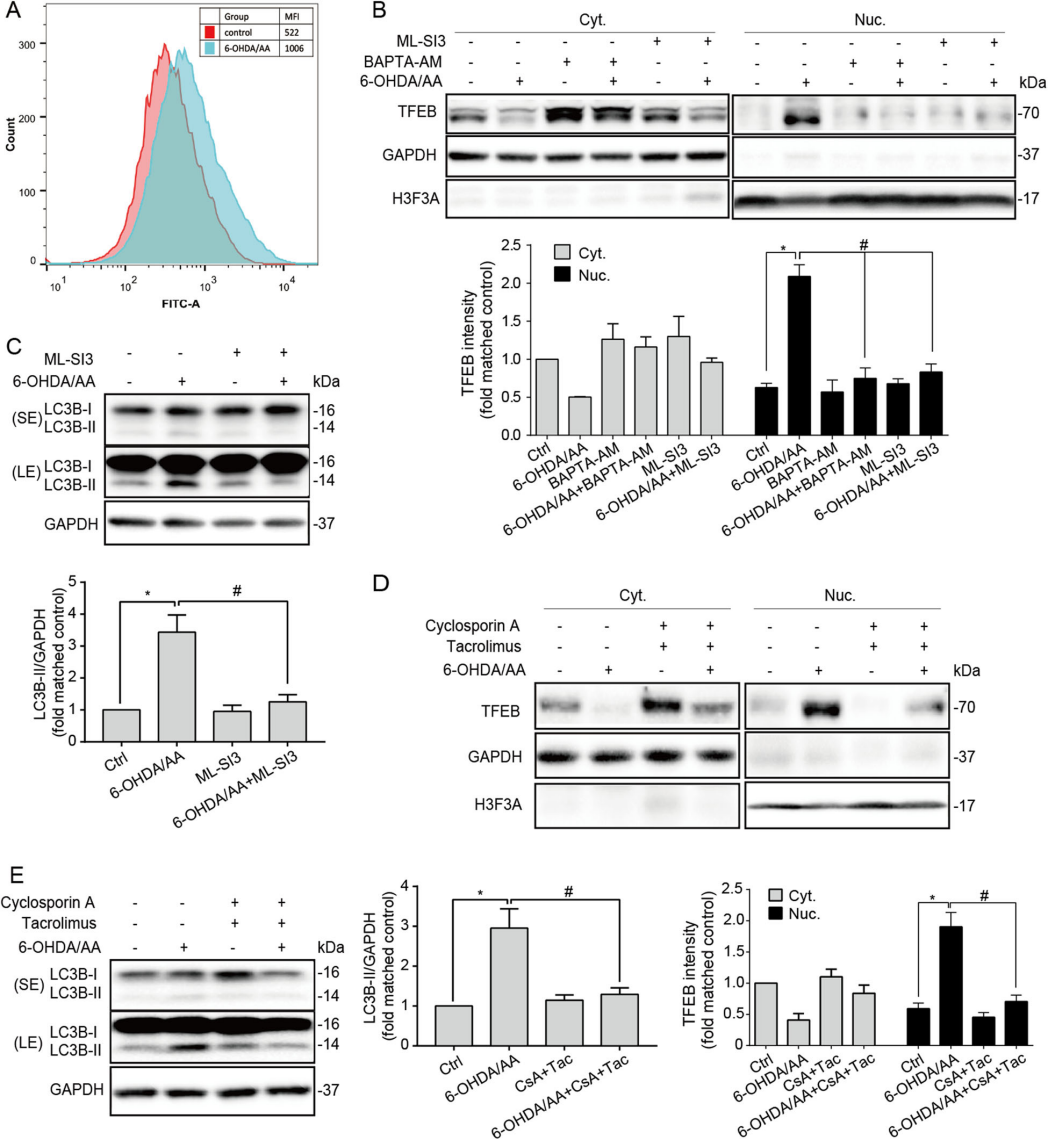
TFEB-mediated autophagy is inducted in a calcium and calcineurin-dependent manner in 6-OHDA/AA models. **a** SH-SY5Y cells preloaded with Fluo-4 AM dissolved in DMEM without phenol red for 30 min, and then treated with 20 μM 6-OHDA/AA for 15 min. After the flow cytometry assay, FlowJo software was used to analyze the mean fluorescence intensity (MFI) in different groups (10,000 cells/group were recorded and analyzed, *n* = 5). **b** SH-SY5Y cells were co-treated with 20 μM 6-OHDA/AA and BAPTA-AM (5 μM) or ML-SI3 (10 μM) for 15 min, and then changed to normal cultured medium containing BAPTA-AM (5 μM) or ML-SI3 (10 μM) for another 6 h incubation (*n* = 5). **c** SH-SY5Y cells were co-treated with ML-SI3 as described in panel (**b**). Data were collected from five independent experiments. **d** SH-SY5Y cells were co-treated with 20 μM 6-OHDA/AA and tacrolimus (Tac. 5 μM) and cyclosporin A (CsA, 10 μM) for 15 min, and then changed to normal cultured medium containing tacrolimus (5 μM) and cyclosporin A (10 μM) for another 3 h incubation. Data were collected from five independent experiments. **e** SH-SY5Y cells were treated as described in panel (**d**). Data were collected from five independent experiments. Above quantifications are shown as mean ± SD. *P < 0.05 vs. Ctrl group; ^#^*P* *<* 0.05 vs. 6-OHDA/AA group.

Very recently, it was reported that lysosomal calcium release induced by oxidative stresses triggers calcineurin-dependent TFEB-nuclear translocation leading to autophagic clearance of damaged mitochondria and removal of excess ROS^22^. We found that 6-OHDA/AA could induce strong mitochondria ROS signal in SH-SY5Y cells (Fig. S2). Co-treatment with Torin1 and/or C1 markedly reduced mitochondrial ROS, which could be blocked by CQ. Furthermore, CQ significantly increased the ROS level induced by 6-OHDA/AA. In addition, we observed a dramatic elevation of intracellular calcium using the Fluo-4 probe in the SH-SY5Y cells treated with 6-OHDA/AA (Fig. 3a). Therefore, we hypothesize 6-OHDA/AA could induce a lysosomal calcium-mediated autophagy. BAPTA-AM, a permeable calcium chelator, was used to chelate intracellular Ca^2+^. As expected, the pretreatment of BAPTA-AM strongly decreased the TFEB nuclear translocation induced by 6-OHDA/AA in SH-SY5Y cells (Fig. 3b). Because lysosomal Ca^2+^ release through MCOLN1 will activate calcineurin, which promotes TFEB nuclear translocation through binding and dephosphorylating it^32^. We next used ML-SI3, a specific inhibitor of MCOLN1 channel, to co-treat SH-SY5Y cells together with 6-OHDA/AA. Remarkably, under this experimental setting, there was only minimal TFEB nuclear translocation (Fig. 3b). Consistently, the increase of LC3B-II induced by 6-OHDA/AA was also greatly blocked by ML-SI3 (Fig. 3c). Furthermore, when calcineurin was inhibited by tacrolimus (FK506) and cyclosporin A (CsA)^32^, the 6-OHDA/AA induced increase of TFEB nuclear translocation (Fig. 3d) and LC3B-II (Fig. 3e) were also greatly blocked. Collectively, these results suggest that autophagy is induced via MCOLN1/calcineurin/TFEB signaling pathway in the 6-OHDA/AA-lesioned PD model.

### Torin 1 and C1 rescues SH-SY5Y cells from 6-OHDA/AA toxicity

**Fig. 4:**
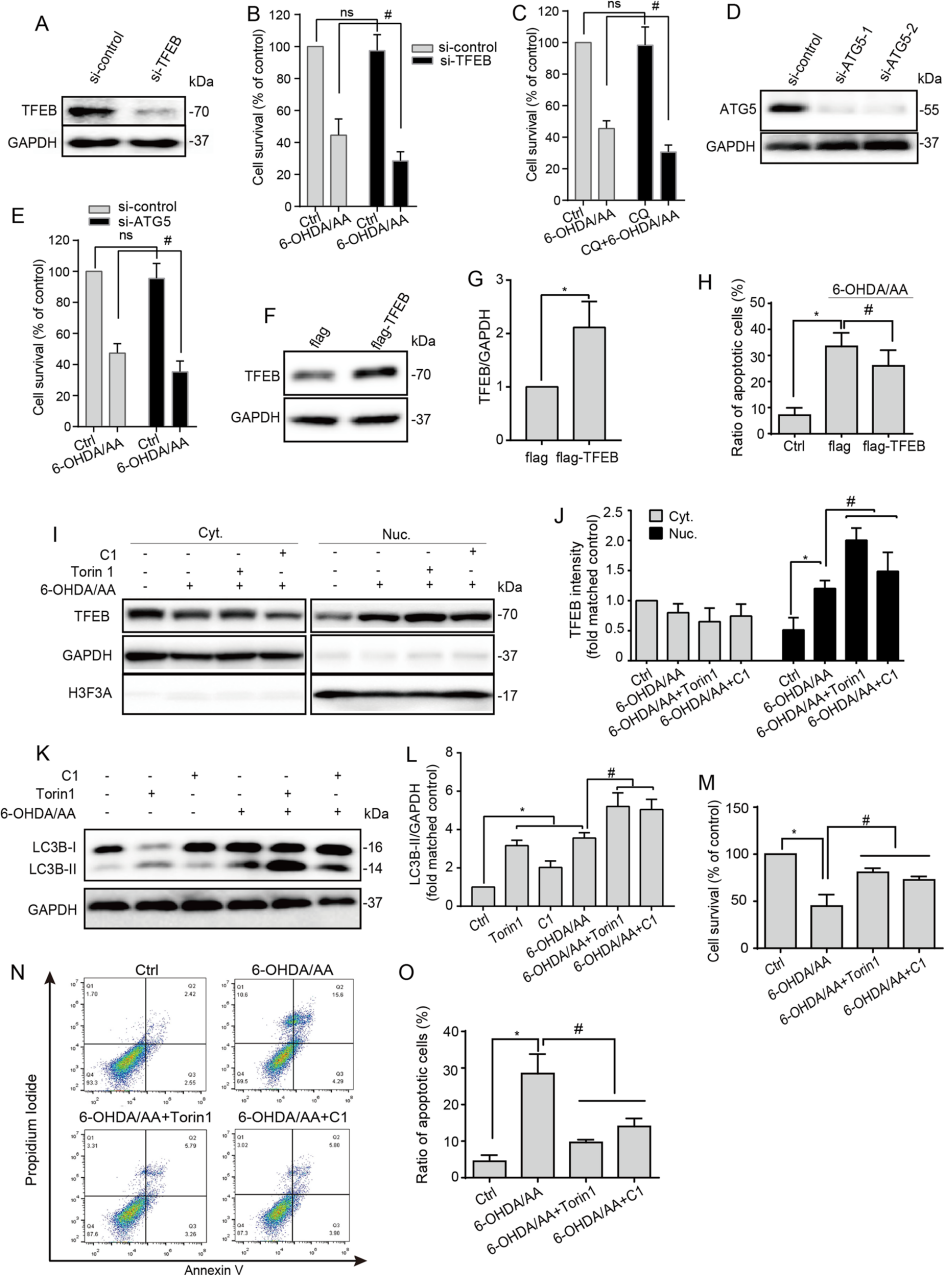
TFEB enhancement rescues SH-SY5Y cells from 6-OHDA/AA toxicity. **a** SH-SY5Y cells were transfected with TFEB siRNA for 48 h, **b** treated as indicated, and then processed for cell survival detection by CCK8 assay. **c** SH-SY5Y cells were treated as indicated with or without CQ (15 μM) for 6 h. **d**, **e** SH-SY5Y cells were transfected with ATG5 siRNA for 48 h, and then treated as indicated for 6 h, and then processed for cell survival detection by CCK8 assay. **f**, **g** SH-SY5Y cells were transfected with flag-TFEB for 24 h and the expression of TFEB detected by WB. **h** Flag-TFEB-overexpressed cells were exposed to 6-OHDA/AA treatment for 15 min and next incubated with normal culture medium for 6 h. Apoptotic cells were detected by PI staining and measured by flow cytometry (*n* = 3). **i**, **j** SH-SY5Y cells were pretreated with C1 (1 μM) for 12 h, then treated with 20 μM 6-OHDA/AA with or without C1 (1 μM) and Torin 1 (1 μM) for 15 min, and then changed with the normal cultured medium with or without C1 (1 μM) and Torin 1 (1 μM) for another 6 h incubation. Data were collected from five independent experiments. **k**, **l** SH-SY5Y cells were treated as described in panel (**i**). **m**–**o** SH-SY5Y cells were treated as described in panel (**c**). The cell survival was detected by CCK8 assay (**m**). The apoptotic cells were detected by Annexin V and Propidium Iodide (PI) double-staining (**n**, **o**). The fluorescence intensities of Annexin V and PI were measured using flow cytometry (*n* = 3). Above quantifications are shown as mean ± SD. *P < 0.05 vs. Ctrl group; ^#^*P* < 0.05 vs. 6-OHDA/AA group.

The ROS-triggered TFEB-mediated autophagy is considered as a cellular compensation action and may play a positive role in cell survival^22^. We found that inhibiting ALP by knocking down TFEB expression (Fig. 4a, b), co-treating with CQ (Fig. 4c), or knocking down ATG5 expression (Fig. 4d, e) significantly increased the toxicity of 6-OHDA/AA. Therefore, we speculated that further activating autophagy function could rescue the cell death induced 6-OHDA/AA toxicity. To confirm this hypothesis, we enhanced TFEB activation to improve autophagy function. Interestingly, over-expression of flag-TFEB in SH-SY5Y cells (Fig. 4f, g) significantly reduced 6-OHDA/AA-induced cell death as measured by PI staining and flow cytometry (Fig. 4h). By co-treating SH-SY5Y cells with Torin 1 or C1, which are chemical enhancers of TFEB activity^29,30^, the TFEB nuclear translocation (Fig. 4I, j) and LC3B-II level (Fig. 4k, l) induced by 6-OHDA/AA were further greatly increased. As expected, co-treatment with Torin 1 or C1 also massively increased the cell viability (Fig. 4m) and prevented cell apoptosis (Fig. 4n, o) in the 6-OHDA/AA toxicity model, as measured by Cell Counting Kit-8 (CCK8) cell viability assay and Annexin V/PI staining, respectively.

**Fig. 5:**
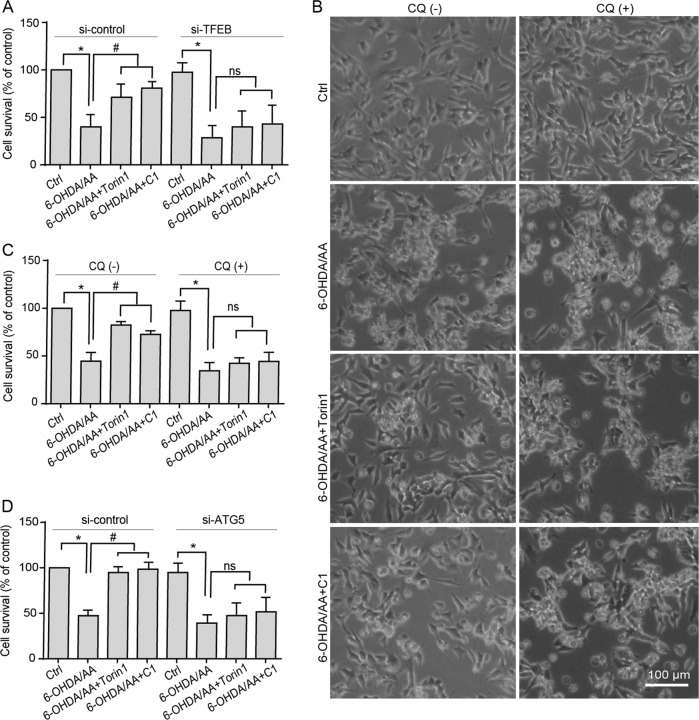
The protective effects of Torin 1 and C1 could be blocked by inhibiting autophagy in the 6-OHDA/AA models. **a** After the downregulation of TFEB by siRNA, the co-treated with Torin 1 or C1 groups showed no significant difference (ns) with 6-OHDA/AA alone group (*n* = 3). **b**, **c** SH-SY5Y cells were treated as indicated with or without CQ (15 μM) for 6 h. At the end of the treatment, the cell morphological changes were observed under phase contract microscope (**b**). **c** The cell survival was determined by CCK8 assay (*n* = 3). **d** SH-SY5Y cells were transfected with ATG5 siRNA for 48 h, and then treated as indicated for 6 h, and then processed for cell survival detection by CCK8 assay (*n* = 3). Above quantifications are shown as mean ± SD. *P < 0.05 vs. Ctrl group; ^#^*P* < 0.05 vs. 6-OHDA/AA group.

To further confirm that the protective effects of Torin 1 or C1 depend on TFEB activation, we knocked down the TFEB expression by using small interfering RNA (siRNA) and found that the protective effects of Torin 1 or C1 were eliminated (Fig. 5a). To confirm the protective effects of Torin 1 or C1 in the 6-OHDA/AA model are autophagy-dependent, the SH-SY5Y cells were firstly co-treated with or without autophagy blocker CQ. Co-treatment with CQ significantly changed the cell morphology as observed under phase contract microscope (Fig. 5b) and decreased cell survival as detected by CCK8 assay (Fig. 5c), indicating the protective effects of Torin 1 or C1 were substantially blocked by CQ. We next silenced the essential autophagy gene ATG5 and found that the protective effects of Torin 1 or C1 were also eliminated (Fig. 5d). Collectively, the data represent evidences that chemical or genetic approaches activating the TFEB-mediated autophagy greatly rescue SH-SY5Y cells from 6-OHDA/AA toxicity.

### Torin 1 and C1 prevent 6-OHDA/AA-induced cytotoxicity in iPSC-derived dopaminergic neurons

**Fig. 6:**
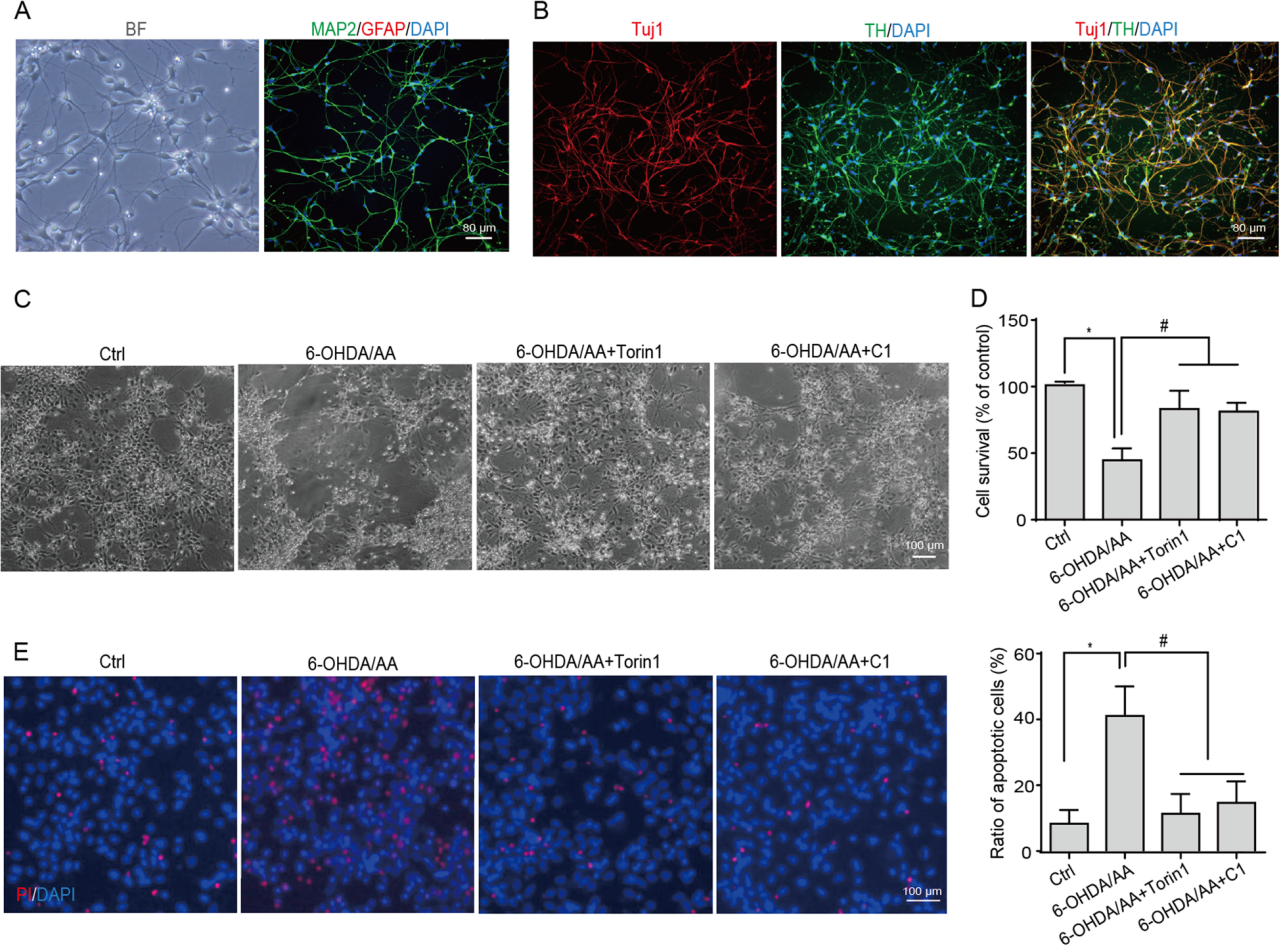
Torin 1 and C1 prevent 6-OHDA/AA-induced cytotoxicity in iPSC-derived dopaminergic neurons. **a** Representative images of immunofluorescence staining with dendritic marker microtubule-associated protein 2 (MAP2, green) and glial fibrillary acidic protein (GFAP, red). **b** Representative images of immunofluorescence staining with neuron-specific class III β-tubulin maker (Tuj1), and dopaminergic neuron marker TH (green). **c** iPSC-derived dopaminergic neurons were pretreated with C1 (0.5 μM) or Torin 1 (1 μM) for 12 h, then treated with 15 μM 6-OHDA/AA with or without C1 (0.5 μM) and Torin 1 (1 μM) for 10 min, and then changed with the dopaminergic maturation medium with or without C1 (0.5 μM) and Torin 1 (1 μM) for another 6 h incubation. The cell morphology was observed under phase contract microscope. **d** The iPSC-derived dopaminergic neurons were treated as indicated in panel (**c**) and cell survival was detected by CCK8 assay. Data were collected from three independent experiments. **e** iPSC-derived dopaminergic neurons were treated as indicated in panel (**c**), and then co-loaded with DAPI (blue) and PI (red). The cell death was determined using a fluorescence microscope. The results are presented as the percentage of PI-positive cells. Data were collected from three independent experiments. Above quantifications are shown as mean ± SD. *P < 0.05 vs. Ctrl group; ^#^*P* < 0.05 vs. 6-OHDA/AA group.

Torin 1 and C1 have been shown to protect neuroblastoma SH-SY5Y cells from 6-OHDA/AA-induced cell death. To extend our finding, the effects of Torin 1 or C1 on dopaminergic neurons treated with 6-OHDA/AA were determined. Dopaminergic neurons were differentiated from a human induced pluripotent stem (iPS) cell line, UC-12-iPSCs. The UC-12-iPSC cell line was derived from urine cells isolated from a healthy donor^33^. We generated dopaminergic neurons as evidenced by the expression of the dendritic marker microtubule-associated protein 2 (MAP2), neuron-specific class III β-tubulin maker (Tuj1), and dopaminergic neuron marker TH (Fig. 6a, b). Cell morphology in 6-OHDA/AA group was seriously impaired (Fig. 6c). Co-treatment with Torin 1 or C1 significantly restored the cell morphology as observed under phase contract microscope (Fig. 6c) and increased the survival of the iPSC-derived dopamine neurons as detected by CCK8 assay (Fig. 6d). In addition, the PI staining revealed that the iPSC-derived dopamine neurons treated with 6-OHDA/AA alone were massively labeled with PI, while Torin 1 and C1 treatment dramatically reduced the ratio of PI-positive cells (Fig. 6e).

### Torin 1 and C1 alleviate 6-OHDA/AA-induced dopaminergic neurons loss in vivo

**Fig. 7:**
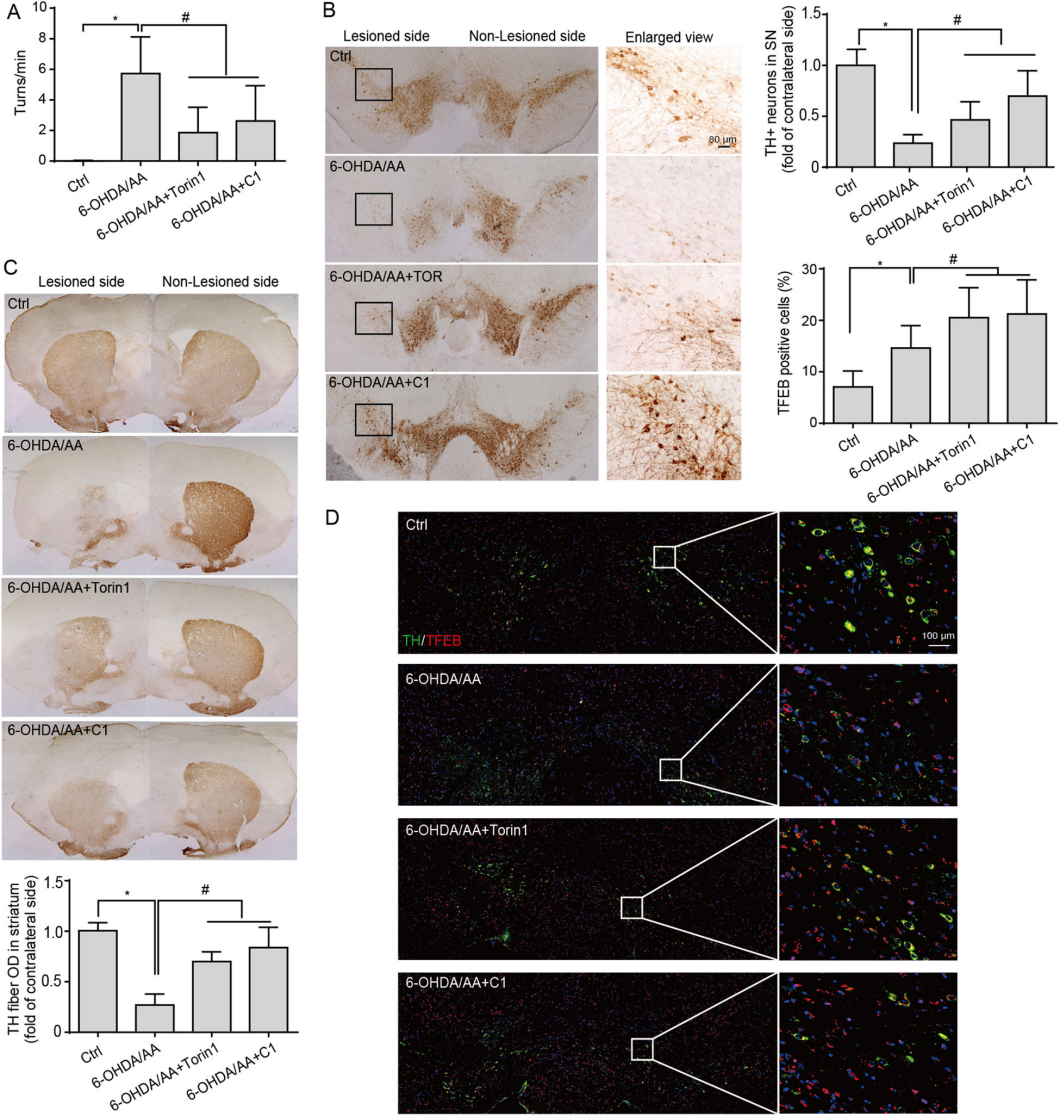
Torin 1 and C1 alleviate 6-OHDA/AA-induced dopaminergic neurons loss in vivo. **a** The mice were pre-intraperitoneally injected with Torin 1 and C1 for 5d, and then received 6-OHDA/AA injection unilaterally. The mice were intraperitoneally injected with or without Torin 1 and C1 for 21 days after 6-OHDA/AA lesion. The behavioral response induced by apomorphine was assessed by counting the rotation number of mice in 20 min. The behavioral response of mice induced by 6-OHDA is represented by the number of rotations to the non-lesioned side per minute (*n* = 12). **b** Transverse sections taken through the SN of mice were immune-stained for TH to assess the extent of dopaminergic lesions induced by 6-OHDA stereotaxic injection with or without Torin 1 and C1 treatment. The Histogram shown the ratio of TH-positive cells in lesioned side to the intact side (*n* = 6). **c** Transverse sections taken through the ST of mice were immune-stained for TH-positive fibers to assess the extent of dopaminergic lesion induced by 6-OHDA/AA stereotaxic injection with or without Torin 1 and C1 treatment. The histogram shows the ratio of TH-positive fibers on the lesioned side to those on the intact side (*n* = 6). **d** The TFEB intracellular localization revealed by immunofluorescence staining with TFEB (red) together with TH (green) in neurons of SNc of mice (*n* = 6). The quantification showed the percent of cells with TFEB nuclear translocation. The results are shown as mean ± SD. *P < 0.05 vs. Ctrl group; ^#^*P* < 0.05 vs. 6-OHDA/AA group.

After confirming the neuroprotective effect of Torin 1 and C1 against 6-OHDA/AA toxicity in SH-SY5Y cells and iPSC-derived dopaminergic neurons, we further tested the compounds on a 6-OHDA/AA-induced mouse PD model. The mice were treated with different concentrations of C1 and Torin 1 via intraperitoneal injection (IP) once a day for 5 days, then the mice received the 6-OHDA/AA stereotaxic injection in the unilateral striatum. After the 6-OHDA/AA injection, C1 and Torin 1 were administered via IP injection once a day for 21 days. Next, the mice were subjected to behavior test and histological analysis. The behavioral assessment revealed significant increase in rotations in the mice unilaterally injected with 6-OHDA after apomorphine hydrochloride (APO) injection, whereas the mice co-treated with Torin 1 or C1 showed decreased rotations induced by APO injection (Fig. 7a). This difference suggested that the behavioral abnormality induced by 6-OHDA/AA was alleviated by subchronic Torin 1 or C1 treatment. TH immunostaining of both SNc and striatum (ST) was also used to measure the dopaminergic toxicity induced by 6-OHDA/AA. The mice treated with the 6-OHDA/AA alone showed more than 65% reduction in the number of the dopaminergic neurons in the lesioned side of the *SNc* compared to the non-lesioned side (Fig. 7b). Surprisingly, co-treatment with Torin 1 or C1 restored the number of TH-positive cells to 80–90% (Fig. 7b). Consistently, the immunohistochemistry staining in ST also revealed a significant preservation of TH-positive fibers of 6-OHDA/AA-treated mice after co-treatment with Torin 1 or C1 (Fig. 7c). In addition, we found that co-treatment with Torin 1 or C1 further increased the number of SNc neurons with striking TFEB accumulation in their nuclei (Fig. 7d) indicating an improvement of TFEB activation in vivo. Taken together, these results show that TFEB activity enhancers Torin 1 and C1 could exert robust neuroprotective effects against 6-OHDA/AA-induced dopaminergic neurons loss in vivo.

### Torin1 and C1 enhance 6-OHDA/AA-induced mitophagy in SH-SY5Y cells

**Fig. 8:**
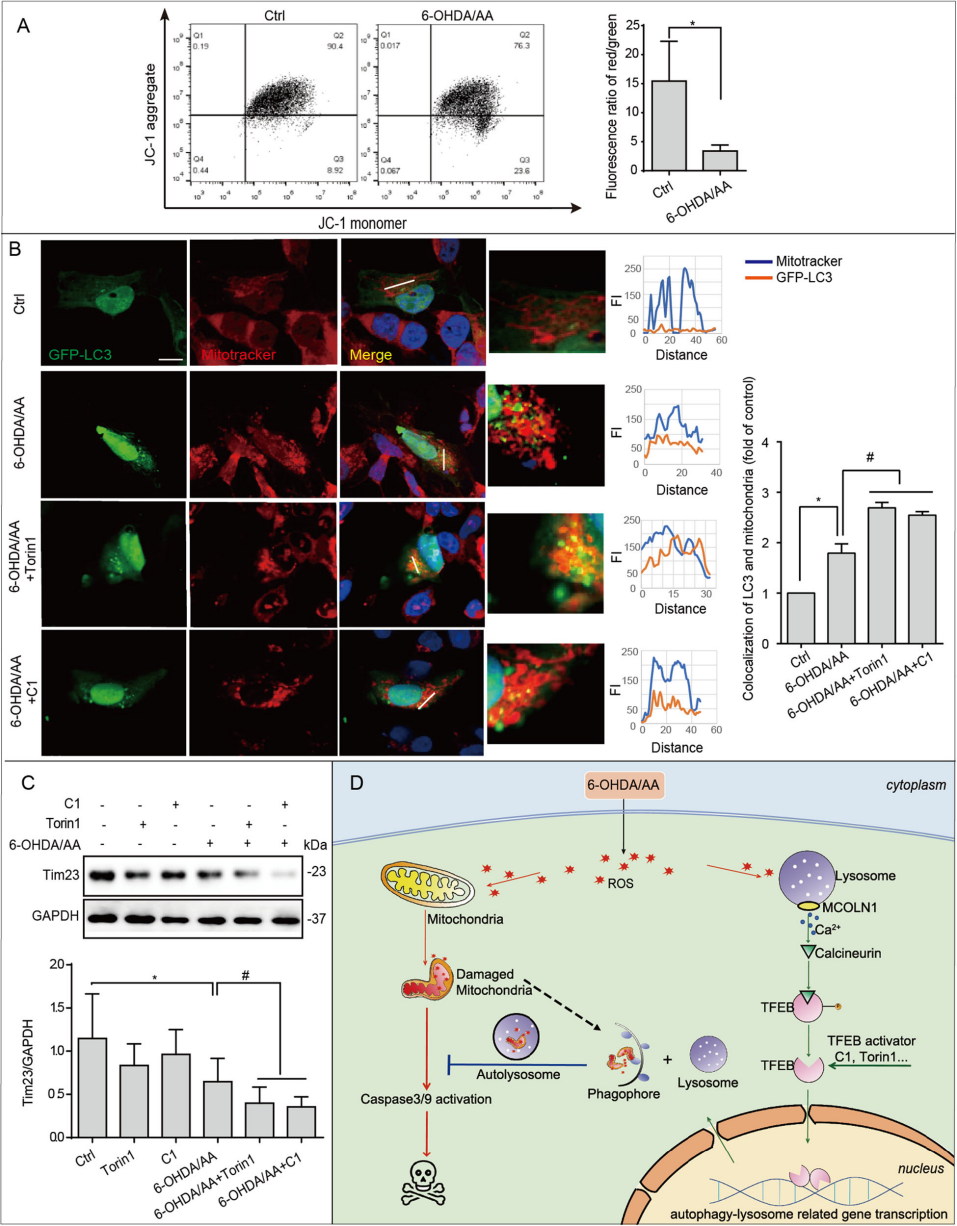
Torin1 and C1 enhanced 6-OHDA/AA-induced mitophagy to eliminate damaged mitochondrial. **a** SH-SY5Y cells were treated with 20 μM 6-OHDA/AA for 15 min, and then changed with the normal culture medium and incubated for 6 h. At the end of treatment, the cells were loaded with JC-1 for 30 min. The fluorescence intensity of 10,000 cells per sample was detected by flow cytometry. **b** SH-SY5Y cells were transiently transfected with GFP-LC3 for 24 h, and then treated with 20 μM 6-OHDA/AA with or without C1 (1 μM, presented for 12 h prior to 6-OHDA/AA incubation) and Torin1 (1 μM) for 15 min, and then changed with the normal culture medium with or without C1 (1 μM) and Torin1 (1 μM) for another 6 h incubation. After treatment, the cells were processed for MitoTracker Red staining to examine the co-localization of mitochondria (red) and GFP-LC3 (green). Representative images are shown. Scale bar, 10 μm. **c** SH-SY5Y cells were subjected to the treatment as indicated and cell lysates were collected and subjected to WB analysis. The results obtained from three independent experiments are shown as mean ± SD. *P < 0.05 vs. Ctrl group; ^#^*P* < 0.05 vs. 6-OHDA/AA group. **d** Proposed model for TFEB-mediated autophagy activation in neuroprotection against 6-OHDA/AA toxicity. The generation of ROS induced by 6-OHDA/AA leads to mitochondria dysfunction, which initiates the apoptotic signaling in cells. But meanwhile, the ROS signal also can be sensed by the calcium channel MCOLN1 on the membrane of lysosome. Release of Ca^2+^ from lysosome leads to calcineurin activation and de-phosphorylation of TFEB. In addition, chemical enhancers like Torin 1 and C1 also can lead to TFEB activation. The activated TFEB translocate to the nucleus to initiate autophagy–lysosome-related genes transcription, which promotes the autophagic clearance of damaged mitochondria and accumulated ROS to rescue cells from death.

Mitochondrial alterations including decreased mitochondrial membrane potential (MMP) and changed mitochondrial morphology have been reported to be associated with 6-OHDA toxicity^34^. Here, we aimed to examine the changes of mitochondrial function and mitophagy in the 6-OHDA/AA model. To do so, we first detected the changes of MMP in SH-SY5Y cells treated with 6-OHDA/AA. As expected, the MMP of cells decreased when they were treated with 6-OHDA/AA for 6 h (Fig. 8a). It has been reported that damaged mitochondria could be removed by mitophagy^35^, an autophagic clearance of damaged mitochondria. To confirm whether damaged mitochondria in the 6-OHDA/AA model could induce mitophagy, we transfected cells with autophagosome marker GFP-LC3, and co-stained with MitoTracker Red to mark mitochondria in SH-SY5Y cells. We found that 6-OHDA/AA treatment for 6 h increased the co-localization of mitochondria with LC3 puncta, interestingly, which could be further improved by co-treatment with Torin1 or C1 (Fig. 8b). TIM23 (also known as TIMM23), an inner membrane protein of mitochondria, has been used to indicate the autophagic clearance of mitochondria^36^. Biochemical analysis revealed that expression level of TIM23 was decreased in SH-SY5Y cells treated with 6-OHDA/AA (Fig. 8c). In addition, co-treatment of Torin1 or C1 markedly decreased the expression of TIM23 compared with 6-OHDA/AA treatment alone (Fig. 8c). Collectively, the data indicate that the neuroprotection of Torin1 and C1 against 6-OHDA/AA-induced toxicity may base on the improvement of autophagic clearance of damaged mitochondria (mitophagy).

## Discussion

In this study, we report novel findings that autophagy is triggered through MCOLN1-calcineurin-dependent TFEB activation in 6-OHDA/AA-lesioned models of PD, and that further promotion of autophagy through TFEB enhancers can greatly rescue SH-SY5Y cells, iPSC-derived and mouse SNc-dopaminergic neurons from 6-OHDA/AA induced neurotoxicity.

6-OHDA is commonly used to model PD for its induction of mitochondrial damage and enhancement of oxidative stress^11^. In the presence of AA, the toxicity of 6-OHDA can be enhanced^15,16^. Even though it has been well-documented that autophagy is induced in the 6-OHDA lesioned models^17–20^, the detailed mechanism underlying it is missed. In addition, an important question remains inconclusive, that is whether 6-OHDA/AA-induced autophagy is a survival mechanism to overcome neurotoxicity or a detrimental factor contributing to cell death?

Very recently, it was reported that lysosomal calcium release induced by oxidative stresses triggers calcineurin-dependent TFEB-nuclear translocation leading to autophagic clearance of damaged mitochondria and removal of excess ROS^22^. In a previous study, we also observed an obvious elevation of intracellular calcium in the SH-SY5Y cells treated with 6-OHDA/AA^16^. These results indicated that a lysosomal calcium release-mediated autophagy as a survival mechanism for cells could be induced in the 6-OHDA/AA toxicity model.

Multiple lines of evidence demonstrated the assumption mentioned above. (i) TFEB, the key regulator of autophagy/lysosome biogenesis, was activated after 6-OHDA/AA treatment both in vivo and in vitro (Fig. 1). (ii) In the 6-OHDA/AA model, autophagy and lysosomal function were promoted (Fig. 2). (iii) The TFEB activation and autophagy induction were in a MCOLN1/calcineurin-dependent manner (Fig. 3). (iv) Chemical or genetic approaches inhibiting ALP increased the toxicity of 6-OHDA/AA (Fig. 4). We noticed a recent study reporting that 6-OHDA induced lysosomal and autophagy dysfunction by inhibition of TFEB activation^21^, which is contrary to our and other groups’ observations^17–19^. Looking into the details of that study, we found that this study showed a transient (within 1 week) reduction of TFEB level in 6-OHDA lesioned mice brain by Western blot. The differences in the detection method and time points may be responsible for the inconsistency in the conclusions. However, considering the heterogeneity of brain tissue, we believe immunohistofluorescence would be a better approach to examine the TEFB activation in the *SNc* area.

Increasing evidence reveals that TFEB, by upregulating the ALP, ameliorates the symptoms of neurodegenerative diseases^8,28,37^. Therefore, in this study, we sought to test whether pharmacological boosting TFEB function will offer protective effects against 6-OHDA/AA-induced toxicity. Our data showed that TFEB overexpression, as well as the TFEB enhancers Torin 1 and C1 are remarkably effective in rescuing SH-SY5Y cells from 6-OHDA/AA-induced toxicity. The mechanism is attributed to the further enhancement of TFEB nuclear translocation and consequent autophagy induction (Fig. 4). The protective effect of Torin 1 and C1 against 6-OHDA/AA-induced toxicity was also confirmed in UC-12-iPSC-derived dopaminergic neurons (Fig. 6) and in a 6-OHDA/AA-lesioned mice PD model (Fig. 7). In addition, chemical or genetic approaches inhibiting autophagic/lysosomal function impairs the protective effects of Torin 1 and C1 against 6-OHDA/AA on SH-SY5Y cells (Fig. 5). All these evidences point to the conclusion that Torin 1 and C1 enhance TFEB nuclear translocation and autophagy induced in 6-OHDA/AA models to exert neuroprotective effects, indicating TFEB activation is a potentially valuable therapeutic strategy against oxidative stress-associated DA neuron degeneration.

An important mechanism underlying the protection of Torin1 and C1 against 6-OHDA/AA-induced toxicity may involve mitophagy induction. Mitochondria quality control is essential for the maintenance of neuron survival. Dysfunctional mitochondria produce excessive ROS and triggers cell death cascades. Recent studies have found that damaged mitochondria undergo mitophagy to prevent further spread of oxidative damage. For instance, a study demonstrates that local mitophagy in distal axons is induced to provide rapid neuroprotection against oxidative stress^38^. Mitophagy induction is also involved in protection against transient ischemic brain injury^39,40^. Here, we show that mitophagy is induced in 6-OHDA/AA model as a stress response (Fig. 8), which is consistent with the fact that mitophagy induction is a compensative mechanism to protect against neuron damage. The treatment with Torin1 and C1 enhanced mitophagy and rescued the cells from 6-OHDA/AA-induced toxicity (Fig. 8), suggesting that mitophagy induction may be a protective mechanism during TFEB/autophagy activation.

In summary, results of the present study indicate that TFEB-mediated autophagy activation during 6-OHDA/AA treatment plays an important role in compensatory protection, and that these protective effects can be increased with autophagy enhancers. Understanding the protective mechanism of TFEB enhancers against 6-OHDA/AA-induced toxicity has important implications in biology and medicine. Modulation of TFEB activity may emerge as a novel anti-PD therapy that not only promotes the clearance of toxic protein aggregates but also prevents oxidative stress-induced DA neuron degeneration. In addition, our results show that the TFEB enhancers Torin 1 and C1 can prevent DA neuron death in mice, pointing even more strongly to the therapeutic potential of TFEB enhancers.

## Materials and methods

### Cell culture and drug treatments

SH-SY5Y cells (ATCC Cat# CRL-2266, RRID:CVCL_0019) were maintained in DMEM/F-12 Media (Gibco) containing 10% fetal bovine serum (Gibco) in a 5% CO_2_ atmosphere at 37 °C. Mycoplasma contamination testing status was negative. The cells were pretreated with C1 for 12 h, and then treated with 6-OHDA/AA in the presence or absence of Torin 1 or C1 for 15 min, after which the medium was removed and replaced with DMEM F12 with or without Torin 1 and C1 for indicated time. After additional incubation, the cells were assessed as described later.

### Dopaminergic neuron differentiation

The UC-12-iPSCs were derived from urine cells isolated from a healthy donor^33^, and were differentiated into dopaminergic neurons as previous described with minor modifications^41,42^. Briefly, stem cells were plated into six-well plates precoated with Matrigel in PSC Neural Induction Medium in the presence of Y27632. The cell culture medium was changed every day for the next 7 days, and then the cells were trypsinizated and plated on dishes precoated with Matrigel at a density of 1.2 × 10^5^ cm^−2^ in PSC neural induction medium with Y27632. The medium was changed every day, and the cells were cultured for dopaminergic differentiation. Briefly, the cells were cultured in six-well plates pre-coated with Matrigel at a density of 2 × 10^5^ cells cm^−2^ in the dopaminergic induction medium (Neural Basal medium with 2% B27, 2 mM Glutamax, 1% nonessential amino acids, 100 U per ml penicillin, and 0.1 mg per ml streptomycin) supplemented with Sonic Hedgehog (SHH, PeproTech, 200 ng mL^−1^), FGF8 (PeproTech, 100 ng mL^−1^), the medium was changed every day up to 10 days. Then the cells were dissociated and seeded into plates pre-coated with poly-l-ornithine/laminin at a density of 2–4 × 10^4^ cells cm^−2^ and incubated for another 10 days in dopaminergic maturation medium consisting of neural basal medium, 2% B27, 1% nonessential amino acids, 2 mM Glutamax, glial cell line-derived neurotrophic factor (GDNF, PeproTech, 20 ng mL^−1^), brain-derived neurotrophic factor (BDNF, PeproTech, 20 ng mL^−1^), 0.2 mM AA (Sigma Aldrich), DAPT (10 nM; Tocris), cAMP (10 μM, Sigma Aldrich), and TGFβ3 (1 ng mL^−1^; R&D)

### Plasmids and siRNA transient transfection

For plasmid transfection, SH-SY5Y cells were transiently transfected with GFP-LC3, GFP-TFEB, flag-TFEB plasmids for 24 h, using lipofectamine^TM^ 3000 according to the protocol from the manufacturer. For the siRNA targeting autophagy gene ATG5 or TFEB was transfected into SH-SY5Y for 48 h, using lipofectamine^TM^ 3000 according to the manufacturer’s protocol.

### MMP determination

Briefly, SH-SY5Y cells were treated as previously described. Then cells were collected by centrifugation, resuspended in JC-1 working buffer and incubated at 37 °C for 15 min. The cells were washed with the buffer and analyzed using a flow cytometer. Totally, 10,000 samples were acquired for the analysis. Red and green populations were subsequently gated for quantification.

### Quantitative real-time PCR (qRT-PCR)

Total RNA was extracted from cells RNeasy Plus Mini Kit (Qiagen, 74134). Reverse transcription was performed using the PrimeScript^TM^ RT reagent Kit (TaKaRa, RR037A). Autophagy and lysosome gene primers were retrieved from a previous study^30^ and synthesized by TSINGKE Biological Technology. Real-time polymerase chain reaction (PCR) was carried out with the SYBR Premix EX Taq^TM^ (TaKaRa) using the Applied Biosystems 7900 HT Fast Real-Time PCR System (Applied Biosystems Inc.). Fold changes were calculated using the DDCT method, and the results were normalized against an internal control (GAPDH). The primer sequences used are listed here:

GAPDH-F, 5′-GTCTCCTCTGACTTCAACAGCG-3′;

GAPDH-R, 5′-ACCACCCTGTTGCTGTAGCCAA-3′;

TFEB-F, 5′- CCTGGAGATGACCAACAAGCAG-3′;

TFEB-R, 5′-TAGGCAGCTCCTGCTTCACCAC-3′;

MAP1LC3B-F, 5′- GAGAAGCAGCTTCCTGTTCTGG-3′;

MAP1LC3B-R, 5′-GTGTCCGTTCACCAACAGGAAG-3′;

SQSTM1-F, 5′- TGTGTAGCGTCTGCGAGGGAAA-3′;

SQSTM1-R, 5′-AGTGTCCGTGTTTCACCTTCCG-3′;

ATG9B-F, 5′-ACCCTGTCAGATGCCATCCTAC-3′;

ATG9B-R, 5′-CCAGTAGCTGAAGAGGTTGCAG-3′;

ATG16L1-F, 5′-CTACGGAAGAGAACCAGGAGCT-3′;

ATG16L1-R, 5′-CTGGTAGAGGTTCCTTTGCTGC-3′;

CTSD-F, 5′-GCAAACTGCTGGACATCGCTTG-3′;

CTSD-R, 5′-GCCATAGTGGATGTCAAACGAGG-3′;

HEXA-F, 5′-GGAGGTCATTGAATACGCACGG-3′;

HEXA-R, 5′-GGATTCACTGGTCCAAAGGTGC-3′.

### Lysosomal pH measurement using LysoSensor and LysoTracker

The intralysosomal PH was estimated using LysoSensor and LysoTracker as the manufacturer’s instructions indicated. SH-SY5Y cells were cultured in black 96-well plates and co-treated with 6-OHDA/AA with or without Torin 1 or C1 for 6 h. Cells were then washed and incubated with 5 μM LysoSensor Yellow/Blue DND-160 in DMEM for 5 min at 37 °C. Light emitted at 535 nm was detected at excitation wavelengths of 340 nm and 380 nm, and the ratio of 340ex/380ex was calculated. For LysoTracker, the cells were treated as indicated and then were stained with LysoTracker for 1 h following manufacturer’s instructions. DAPI was used to visualize the nuclei. The fluorescence intensity was observed under a confocal microscope (Leica TCS SP8, Leica Microsystems); representative cells were selected and photographed.

### Western blot

Cells were lysed in RIPA buffer (Beyotime Biotechnology) containing 50 mM Tris-HCl, 1% NP40, 0.35% DOC, 150 mM NaCl, 1 mM EDTA, 1 mM EGTA, supplemented with completed protease phenylmethylsulfonyl fluoride (PMSF) and phosphatase inhibitor cocktail. The lysates were denatured in loading buffer and separated by 8–12% sodium dodecyl sulfate (SDS) polyacrylamide gel electrophoresis. The protein was transferred onto polyvinylidene fluoride (PVDF) membrane and blocked with 5% nonfat milk in Tris-buffer saline containing 0.05% Tween-20. The proteins were blotted with the antibodies indicated overnight. The blots were then incubated with secondary antibodies at room temperature for about 1 h. The protein signals were visualized by ECL and detected in an image reader.

### Immunofluorescence staining

SH-SY5Y cells were seeded on a cover glass slide chamber overnight. After the designated treatment, the cells were washed with phosphate-buffered saline (PBS), and then fixed with 4% paraformaldehyde for 15 min at room temperature. Then the cells were permeabilized with 0.2% Tritonx-100 in PBS for 20 min. The cells were washed with PBS and blocked with 5% sheep serum in PBS for 1 h, and then incubated with primary antibodies overnight at 4 °C. Secondary antibodies were incubated for another 2–3 h. DAPI was used to visualize the nuclei. The cells were observed using a confocal microscope (Leica TCS SP8, Leica Microsystems).

### Measurement of cell viability using CCK8 assay

Cell viability was determined by CCK8 assay following manufactory’s manual. Cells were plated at the density of 10^4^ cells per well in 24-well plates in 500 μl DMEM. After the indicated treatment, CCK8 reagents (Beyotime Biotechnology) were added to the culture medium for 2–3 h. Then the optical densities of the wells were read on a microplate reader at 450 nm. The percentage of viability was calculated by the ratio of experimental cells to normal cells.

### Annexin V/PI staining assay

The apoptosis of the SH-SY5Y cells was determined with Annexin V/propidium iodide (PI) staining kit by flow cytometry. Staining was performed based on the kit instructions. Briefly, after the indicated treatment, cells were collected and stained with Annexin V at room temperature for 30 min, then PI was added to each sample prior to flow cytometry analysis. Totally 10,000 cells were collected and analyzed using the FACS cytometer (BD Bioscience).

### Subcellular fractionation

Cells were plated on 10 cm dishes until the density reached 70–80%; then, the cells were treated as indicated. At the end of the treatment, the cells were washed with PBS twice and lysed in 500 μl ice-cold hypotonic buffer (50 mM Tris-HCl, 137.5 mM NaCl, 0.5% Tritonx-100, 10% glycerol, 5 mM EDTA, supplemented with completed protease PMSF and phosphatase inhibitor cocktail) for 15 min and spun at 4 °C for 5 min at 500*g*. The supernatant (cytoplasmic fraction) was transferred to a new tube. Totally, 100 μl ice-cold hypotonic buffer was added to the pellet and centrifuged at 4 °C for 5 min at 500*g*. Next, 100 μl 2% SDS in RIPA supplemented with protease PMSF and phosphatase inhibitor cocktail was added to the rest of the pellet and sonicated on ice for 5 min. The rest of the homogenate was spun at 4 °C for 30 min at 12,500 rpm. The concentration of protein was measured with BCA reagent (Beyotime Biotechnology). The samples were analyzed by Western blot.

### Animals and treatments

#### Animal ethics

A 12-week-old C57BL/6J female mice (22–25 g) were used. All mice were kept under standard laboratory conditions. The experiment procedures were approved by the University of Macau Animal Research Ethics Committee with an ethical No. UMARE-051-2017, following the guidelines for the care and use of Laboratory animals^43^.

#### Animal treatments

Totally, 48 mice were randomly separated into 4 experimental groups: control (or sham, drug vehicle, 10% Solutol HS 15); 6-OHDA/AA (drug vehicle, 10% Solutol HS 15), Torin 1 (5 mg kg^−1^, 10% Solutol HS 15); C1 (10 mg kg^−1^, 10% Solutol HS 15), with 12 mice in each group. The sample size in each group is enough for the behavioral test and the following biochemical analysis. Mice were pretreated with drugs (Torin 1 or C1) or drug vehicle by intraperitoneal administration daily for 5 days before lesion.

After 5 days pretreatment, all the mice received a unilateral injection of 6-OHDA (12 μg dissolved in 2 μl 0.15% AA/PBS) or 2 μl 0.15% AA/PBS into striatum. The stereotactic co-ordinates used for the lesion were: AP + 0.5 mm, LM + 2.4 mm, DV + 3.5 mm. After lesion, the mice received drugs or drug vehicles daily for another 21 days. In the end, all the mice were sacrificed under anesthesia by transcardial perfusion with ice-cold PBS and 4% paraformaldehyde.

#### Behavioral tests

Twenty-one days after right intranigral stereotaxic injection of 6-OHDA, all the animals were subjected to the rotational test before the sacrificing. Briefly, mice were injected with 5 mg kg^−1^ APO and placed in a smooth-sided beaker. After 5 min the number of contralateral rotations was recorded for the next 20 min. The data from a mouse accidentally died would be excluded from the analysis.

#### Immunohistochemistry and TH neuron counts

The whole frozen barin tissues that containing substantia nigra and striatum were cut at 30 μm thickness and stained with TH antibody. Briefly, the sections were washed with PBS three times, quenched with 3% H_2_O_2_, and then incubated with 5% bovine serum albumin (BSA) in 0.2% TritonX-100 for 1 h at room temperature. After permeabilization, the sections were incubated with rabbit anti-TH antibody in 5% BSA (1:500, Pel-Freez Biologicals, Arkansas) at 4 °C overnight. The sections were washed with PBS three times, subsequently incubated in the secondary antibody for 1 h at room temperature, and finally incubated with streptavidin–biotin–horseradish peroxidase complex. TH immunoreactivity was visualized using a DAB kit. The neuronal counts were performed by using a stereomicroscope (BX51, Olympus Corp. Japan). At ×10 magnifications, 6 position-matched sections containing SNc per mouse were selected and manually counted TH-positive neurons by an operator blinded to the drug administration. Above neuronal counting process was performeded in six mice per group and the mean of TH-positive neurons represented the livability of dopaminergic neurons. TH fiber OD in striatum was analyzed with ImageJ software. The values were calculated as the ratio of the ipsilateral side to the contralateral side.

### Materials

The chemicals used in our experiments were: 6-hydroxydopamine hydrobromide (6-OHDA, Sigma, H8523); AA (Aladdin, A103540); Torin 1 (LC Laboratories, T-7887); C1 (synthesized according to our previous study^44^; CQ (Sigma, C6628); LysoTracker Red DND-99 (Thermo Fisher, M22425); LysoSensor Yellow/Blue DND-99 (Thermo Fisher, L7545; Cyclosporin A (MedchemExpress, HY-B0579); MitoSOX^™^ Red Mitochondrial Superoxide Indicator (Molecular Probes, Inc., M36008); Tacrolimus (MedchemExpress, HY-13756); BAPTA-AM (MedchemExpress, HY-100545). The antibodies used in our experiments were: LC3B (Novus Cat# NB100-2220, RRID:AB_10003146); ATG5 (Cell Signaling Technology Cat# 12994, RRID:AB_2630393); LAMP-1 (Cell Signaling Technology Cat# 9091, RRID:AB_2687579); Cathepsin D (Santa Cruz Biotechnology Cat# sc-6486, RRID:AB_637896); Cathepsin B (Santa Cruz Biotechnology Cat# sc-365558, RRID:AB_10842446); TFEB (Bethyl Cat# A303-673A, RRID:AB_11204751); Histone H3/H3F3A (Cell Signaling Technology Cat# 4499, RRID:AB_10544537); GAPDH (Cell Signaling Technology Cat# 5174, RRID:AB_10622025); P-S6K (T389), P-S6K (T371), mTOR and p-mTOR (Anti-mTOR Substrates Sampler Kit, Cell Signaling Technology Cat# 9862, RRID:AB_10696885); TH (Pel-Freez Biologicals Cat# P40101-0, RRID:AB_461064). Tim23 (BD Biosciences, 611222). The siRNA used in our experiments: ATG5 siRNA, TFEB siRNA (GenePharma Co., Ltd.). The plasmids used in our experiments were: GFP-LC3-RFP (RRID:Addgene_117413), GPF-TFEB (RRID:Addgene_38119).

### Statistical analysis

Operator and data analysis were blinded. Data were presented as mean ± SD. Statistical analysis was performed with GraphPad Prism software (Version 7.00, GraphPad Software, Inc.). A two-tailed Student’s *t* test was used when comparing data from two groups. One-way ANOVA was followed by the Student–Newman–Keuls test (post hoc tests were conducted only if *F* was significant and there was no variance inhomogeneity) for comparisons between multiple groups. Data normalization (e.g., in the Western blot analysis) was performed to control for sources of variation of baseline parameters. These data were subjected to nonparametric Mann–Whitney *U*-test. For all analyses, statistical significance was considered when probability value of *P* < 0.05.

## Supplementary information

CDDis-19-2491RRR-author-contribution

MS-CDDIS-19-2491RRR-Reporting Checklist

Supplementary figure legends

Figure S1

Figure S2
