# Supplementary material for: Pharmacological enhancement of TFEB-mediated autophagy alleviated neuronal death in oxidative stress-induced Parkinson’s disease models
Source: Cell Death Dis. 2020 Feb 18;11(2):128. doi: 10.1038/s41419-020-2322-6 (PMC7028954; doi:10.1038/s41419-020-2322-6)
Supplement: Supplementary file 1 — CDDis-19-2491RRR-author-contribution [file 41419_2020_2322_MOESM1_ESM.pdf]

# DECLARATION OF CONTRIBUTIONS TO ARTICLE

**ADMC**

| Manuscript Number: | Journal Name: | |
|---|---|---|
| CDDIS-19-2491RRR | *Cell Death & Disease* | (the 'Journal') |

Proposed Title of the Contribution:

Pharmacological enhancement of TFEB-mediated autophagy alleviated neuronal death in oxidative stress-induced Parkinson's disease models (the 'Contribution)

Author(s):

Xu-Xu Zhuang, Sheng-Fang Wang, Yuan Tan, Ju-Xian Song, Zhou Zhu, Zi-Ying Wang, Ming-Yue Wu, Cui-Zan Cai, Zhi-Jian Huang, Jie-Qiong Tan, Huan-Xing Su, Min Li, Jia-Hong Lu (the 'Authors')

For all *CDDis* articles, each person named as an author in the published version must be able to show he or she has contributed substantially to the article.

Authorship credit should be based on 1) substantial contributions to conception and design, acquisition of data, or analysis and interpretation of data; 2) drafting the article or revising it critically for important intellectual content; and 3) final approval of the version to be published. Authors should meet conditions 1, 2 and 3.

Any person who cannot be shown to have made a substantial contribution to the article cannot be listed as an author in the final version. The name of any person who is deemed to have made a minor contribution can, however, appear in the Acknowledgments section of the article.

Please complete the table below to indicate the contributions of all named authors to the manuscript.

| Author Full Name: | Specification of Contribution to the Manuscript: |
|---|---|
| Xu-xu Zhuang | acquisition and analysis of data; drafting and revising the MS |
| Sheng-Fang Wang | acquisition and analysis of data; drafting and revising the MS |
| Yuan Tan | acquisition and analysis of data |
| Ju-Xian Song | experiment conception and design; revising the MS |
| Zhou Zhu | analysis and interpretation of data |
| Zi-Ying Wang | analysis and interpretation of data |
| Ming-Yue Wu | analysis and interpretation of data |
| Cui-Zan Cai | analysis and interpretation of data |
| Zhi-Jian Huang | acquisition and analysis of data |
| Jie-Qiong Tan | experiment conception and design |
| Huan-Xing Su | experiment conception and design; final approval of the version to be published |
| Min Li | experiment conception and design; final approval of the version to be published |
| Jia-Hong Lu | experiment conception and design; revising the MS; final approval of the version to be published |

Please complete the table below to indicate the contributions of all named authors to the figures.

Figure 1:

XXZ generated the Western blot data in panel A and assembled the figure; SFW generated the data and prepared panel B-D.

Figure 2:

SFW generated the data and prepared panel A, C, E, F, and G; XXZ generated the data in B, D, and H, and assembled the figure

Figure 3:

XXZ generate the data in panel A-E and assembled the figure

Figure 4:

XXZ generated the data in panel B, C, E-G, I-L and assembled the figure; SFW generated the data in panel A, D, H, M-O.

Figure 5:

SFW generated the data and prepared panel A-F; XXZ assembled the figure

Figure 6:

YT generated the data and prepared panel A, B; ZJH generated the data prepared panel C-E; SFW assembled the figure.

Figure 7: SFW generated the data and prepared panel A-D; XXZ assembled the figure.

Figure 8: SFW generated the data and prepared panel A-D; XXZ assembled the figure; XXZ drew the schematic diagram.

| Signed for and on behalf of the Author(s): | Print Name: | Date: |
| --- | --- | --- |
| [signature] | Jia-Hong Lu | 01/02/2020 |
