# Supplementary material for: Pharmacological enhancement of TFEB-mediated autophagy alleviated neuronal death in oxidative stress-induced Parkinson’s disease models
Source: Cell Death Dis. 2020 Feb 18;11(2):128. doi: 10.1038/s41419-020-2322-6 (PMC7028954; doi:10.1038/s41419-020-2322-6)
Supplement: Supplementary file 3 — Supplementary figure legends [file 41419_2020_2322_MOESM3_ESM.docx]

**SUPPLEMENTARY FIGURE LEGENDS**

**SUPPLEMENTARY FIGURE 1**

(A) SH-SY5Y cells were treated with different concentrations of 6-OHDA-only or 6-OHDA/ ascorbic acid (AA) for 15 min, and then drug-containing DF12 medium was replaced with normal DF12 medium for a 24 h incubation. Cell viability was assessed by CCK-8 assay. (B) SH-SY5Y cells were treated with vehicle (culture medium containing 0.15% AA, control group, Ctrl for short), 20μM 6-OHDA in normal culture medium, and 20μM 6-OHDA in culture medium containing 0.15% AA (6-OHDA/AA) for 15 min, and then replaced with normal culture medium for 6h incubation. The levels of TFEB in the cytosolic (Cyt.) and nuclear (Nuc.) fractions were detected by Western blot. GAPDH and H3F3A were used as loading controls of the cytosolic and nuclear fractions, respectively. (C) TFEB intensity in different groups were analyzed (n=4). (D) SH-SY5Y cells were treated as described in panel (B). The upper band showed the short-time exposure (LE) of LC3B while the middle one showed the long-time exposure (LE) of LC3B. (E) The ratio of LC3B-II/LC3B-I in diferent groups was quantified (n=4). (F, G) SH-SY5Y cells were treated with 20μM 6-OHDA/AA as described in panel (B) for 6h. After the treatment, cells were stained with LysoTracker Red DND-99 (50 nM) for 30 min and then were checked under a confocal microscope. Lysosome number (F) and size (G) were analyzed with ImageJ software (n=3). Above quantifications are shown as mean ± SD. * or #, P < 0.05.

**SUPPLEMENTARY FIGURE 2**

**Mitochondrial ROS is significantly induced in 6-OHDA/AA model.** SH-SY5Y cells with indicated treatments for 3h were loaded with 5μM MitoSOX^TM^ reagent working solution for 10min at 37℃ and then incubated with DAPI. Images were acquired by using In Cell Analyzer 2000. Quantification of MitoSOX Red fluorescent intensity (FI) in relation to DAPI FI were performed with ImageJ (n=5). Data are shown as mean ± SD. *, P < 0.05 vs. Ctrl group; #, P < 0.05 vs. 6-OHDA/AA group.
