# Supplementary figures and images for: Pharmacological enhancement of TFEB-mediated autophagy alleviated neuronal death in oxidative stress-induced Parkinson’s disease models
Source: Cell Death Dis. 2020 Feb 18;11(2):128. doi: 10.1038/s41419-020-2322-6 (PMC7028954; doi:10.1038/s41419-020-2322-6)

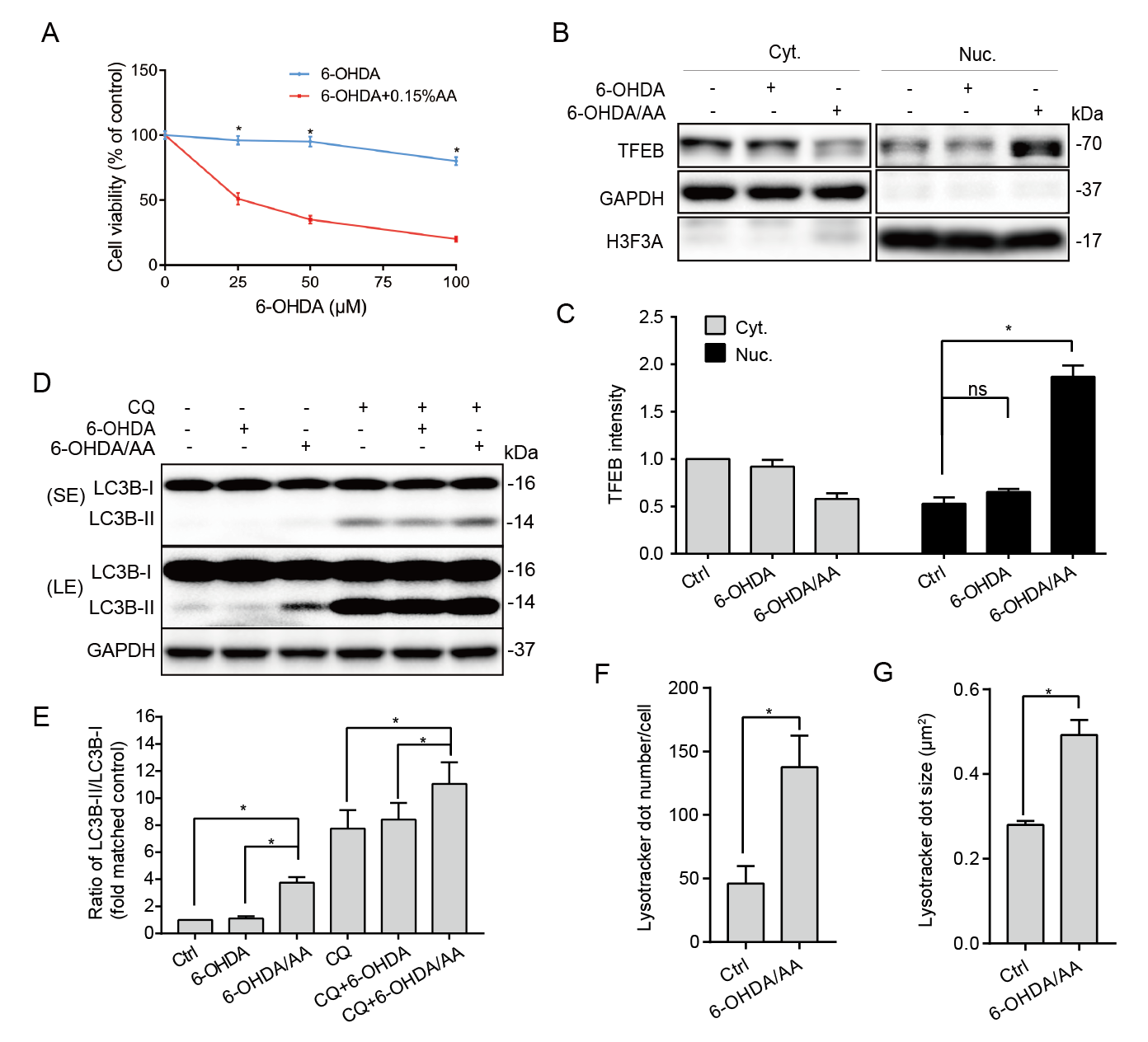

Supplement: Supplementary file 4 — Figure S1 [file 41419_2020_2322_MOESM4_ESM.png]

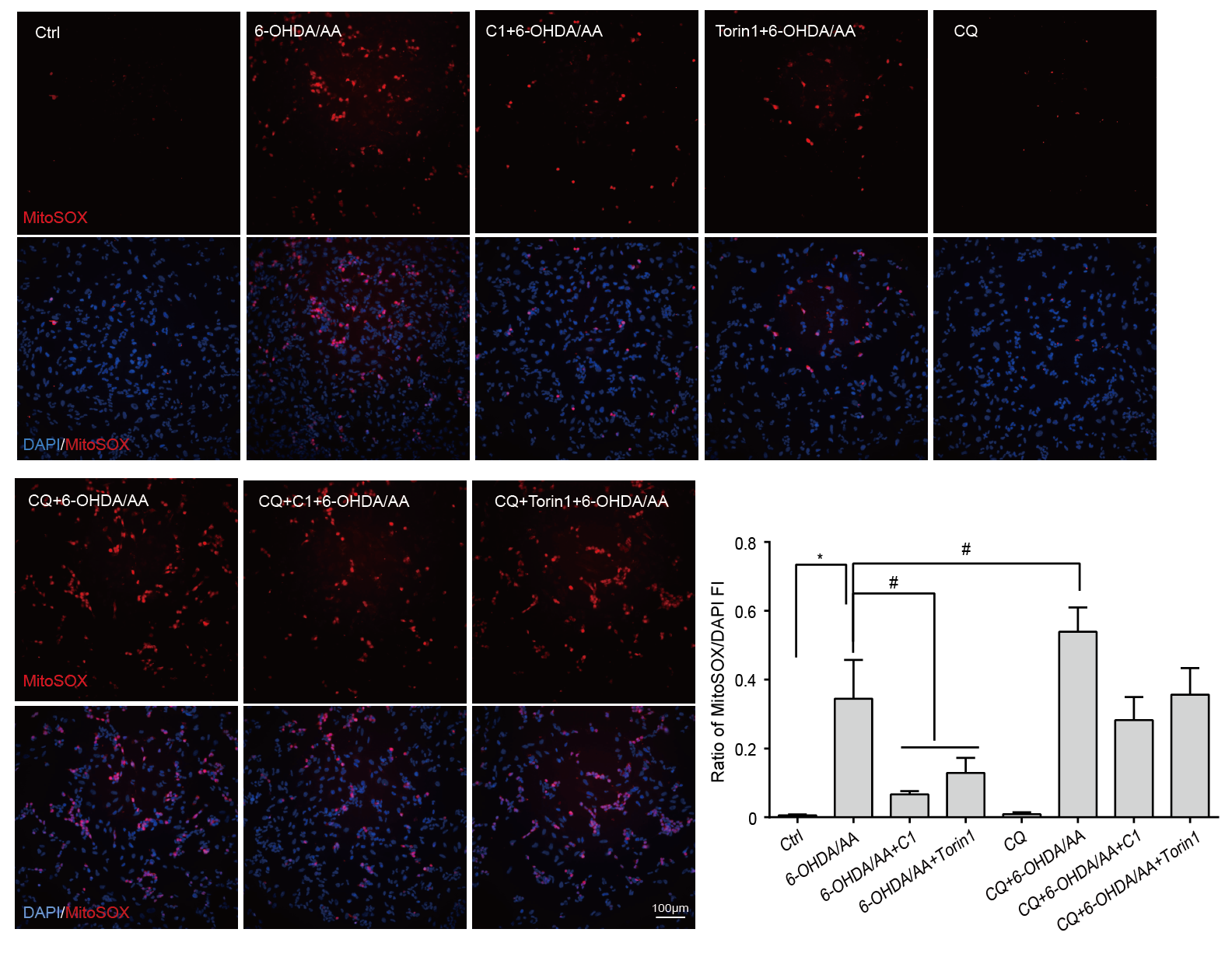

Supplement: Supplementary file 5 — Figure S2 [file 41419_2020_2322_MOESM5_ESM.png]
